# A Full Technological Traceability System for Extra Virgin Olive Oil

**DOI:** 10.3390/foods9050624

**Published:** 2020-05-13

**Authors:** Simona Violino, Federico Pallottino, Giulio Sperandio, Simone Figorilli, Luciano Ortenzi, Francesco Tocci, Simone Vasta, Giancarlo Imperi, Corrado Costa

**Affiliations:** Consiglio per la Ricerca in Agricoltura e l’Analisi dell’Economia Agraria (CREA)—Centro di Ricerca Ingegneria e Trasformazioni Agroalimentari, Via della Pascolare 16, 00015 Monterotondo (Rome), Italy; simonaviolino@hotmail.com (S.V.); federico.pallottino@crea.gov.it (F.P.); giulio.sperandio@crea.gov.it (G.S.); simone.figorilli@crea.gov.it (S.F.); luciano.ortenzi@crea.gov.it (L.O.); francesco.tocci@crea.gov.it (F.T.); simone.vasta@crea.gov.it (S.V.); giancarlo.imperi@crea.gov.it (G.I.)

**Keywords:** EVOO, supply chain, RFID, infotracing, blockchain, Arduino, made in Italy

## Abstract

The traceability of extra virgin olive oil (EVOO) could guarantee the authenticity of the product and the protection of the consumer if it is part of a system able to certify the traceability information. The purpose of this paper was to propose and apply a complete electronic traceability prototype along the entire EVOO production chain of a small Italian farm and to verify its economic sustainability. The full traceability of the EVOO extracted from 33 olive trees from three different cultivars (Carboncella, Frantoio and Leccino) was considered. The technological traceability system (TTS; infotracing) consists of several open source devices (based on radio frequency identification (RFID) and QR code technologies) able to track the EVOO from the standing olive tree to the final consumer. The infotracing system was composed of a dedicated open source app and was designed for easy blockchain integration. In addition, an economic analysis of the proposed TTS, with reference to the semi-mechanized olive harvesting process, was conducted. The results showed that the incidence of the TTS application on the whole production varies between 3% and 15.5%, (production from 5 to 60 kg tree^−1^). The application at the consortium level with mechanized harvesting is fully sustainable in economic terms. The proposed TTS could not only provide guarantees to the final consumer but could also direct the farmer towards precision farming management.

## 1. Introduction

One of the main problems in the agri-food sector is to define tools useful to determine the origin of products and raw materials in order to ensure their traceability [1]. Consumer demand is indeed increasingly oriented towards the purchase of certified food products; therefore, geographical identification becomes an important tool to guarantee consumer safety. This is especially true for extra virgin olive oil (EVOO), the quality of which is strongly linked to the cultivars used and the environmental conditions of the growing area [2]. In this framework, food traceability acquires a major relevance. EVOO traceability is a relevant process that allows for the identification of the varieties of olives. Moreover, it is not only crucial for establishing the olive oil origin, but it is fundamental for the protection of consumers from fraud [3]. For this reason, tools to verify the traceability process are needed to control the quality of virgin olive oil introduced on the market [4]. Furthermore, the traceability of EVOO is of growing interest for producers, as it improves the supply chain optimization, the competitiveness among the producers and the prevention of mislabeling of the olives’ geographical origins and varieties. Thus, it guarantees that the right information reaches the consumers [5].

“Traceability” can be defined as the ability to follow the movement of a product in each phase, from production, processing and distribution, monitoring the history, origin of materials and parts and processing until its distribution. EVOO represents a key ingredient of the Mediterranean diet, with a significant role for international food commerce of many countries, including Italy [6]. Traceability is not only crucial for EVOO origin certification but is fundamental for consumer protection against fraud [3]. To protect and preserve one of the most famous Italian food products, constant efforts and continuous progress are required to combat new fraudulent practices [7]. In order to promote and preserve the geographic identification of EVOO, the European Commission applies two certifications, namely the protected designation of origin (PDO) and the protected geographical indication (PGI) [6]. These certification systems are based on product labelling. However, the sole use of food labels is not enough to ensure product authenticity, quality and safety [8]. Therefore, to prevent fraud and counterfeiting, along the entire EVOO production chain, it is necessary to implement traceability procedures that are currently available [9].

As reported by Papetti et al. [10], the process that keeps records and reports the trail of an input from outfitter to customers is defined as an infotracing system. Infotracing systems include on one side strategies to safeguard the information flow authenticity, and on the other it allows consumers to be efficiently protected against fraud. Consumers can indeed retrieve information on the product by reading a physical label on the packaging or by using a digital application. Generally, it is possible to obtain information about traceability along the food supply chain by using different kinds of smart tags such as radio frequency identification (RFID), near field communication (NFC), barcodes, etc. Due to its versatility and reading distance, RFID technology is normally used for product identification and for traceability in the agri-food sector to guarantee food safety and quality. Many authors describe the advantages coming from the use of RFID within food traceability systems. As reported by Regattieri et al. [11], RFID technology is suitable for food supply chain, as it does not require physical contact for the reading phase and is both fast and completely automated. 

Hsu et al. [12] applied an RFID-enabled traceability system for a live fish supply chain. Nguyen et al. [13], instead, proposed the use of passive UHF RFID tags to detect contamination and quality along the fresh meat chain. Another study evaluated the implementation of RFID to an anti-counterfeit mechanism in selected wine production environments (Bernardi et al. [14]). In this case, a RFID tag allowed the reseller and final user to verify whether the bottle was original or not. Papetti et al. [10] proposed the integration of an electronic RFID tracing system for a single product of a typical Italian cheese prepared with buffalo milk.

RFID is considered to be a successor of the barcode with foreseen expansion not only in the agri-food sector, but also in industrial sectors due to its environmental monitoring capabilities (temperature, relative humidity and luminosity), namely through wireless sensor networks (WSNs) and wireless sensor technology (WST). RFID systems are composed of the following: (1) a reader or interrogator for wave emission and reception upon backscattering from the RFID tags; (2) several tags that can be equipped with a chip for unique identification (using an electronic product code) or/and a rewritable memory for data storage, sensors for environmental monitoring and an antenna used for communications; and (3) a software infrastructure and a host computer for data managing and analysis [15]. In addition to traceability benefits, RFID systems produce logistic advantages that are automatable and readable by logistic platforms [6].

Additionally, as reported by Figorilli et al. [16], an infotracing system can integrate information related to the quality of the product with those related to its traceability (physical and digital documents (RFID architecture)), taking advantage of an online information system whose steps (transactions) can be protected to proof eventual alteration (e.g., using a blockchain system). Blockchain is a distributed database of records in the form of encrypted “blocks” (smaller datasets), or a public ledger of all transactions or digital events that have been played out and shared among participating parties, which can be verified at any time in the future [17]. Each transaction in the public master is verified by consensus of a majority of participants in the system. Once entered, information can never be deleted. The blockchain contains a certain, verifiable record of every single transaction ever made, and its blocks can be used to coordinate an action or verify an event [18].

The aim of this paper is to apply a prototype of full electronic traceability along the entire EVOO production chain of a small producer and to verify the economic sustainability of its implementation within a production system. The prototype was built using different technologies (RFID and QR code) and applied (in a central Italy orchard) to trace the EVOO from standing olive tree to final consumers. The infotracing architecture, also composed by dedicated open source apps, was designed for an easy integration of a blockchain platform. The work also explores the economic sustainability of its implementation.

## 2. Materials and Methods 

### 2.1. EVOO Traceability

The EVOO produced by thirty-three olive trees was followed along the whole supply/processing chain from standing trees to consumer. The olive trees and the olive oil mill production site under study were on the Ponzani Antonio farm, in Montorio Romano (Central Italy; N 42.1395917; E 12.77265). The olive orchard, located in a hilly area, is characterized by a ground with an average slope of 35%, and an irregular planting layout with average distances between the trees of 10.2 × 8.9 m, equal to an average density of about 110 trees per hectare. The harvest was conducted between 7 November and 27 November. The olive pressing was conducted on 27 November.

**Figure 1:**
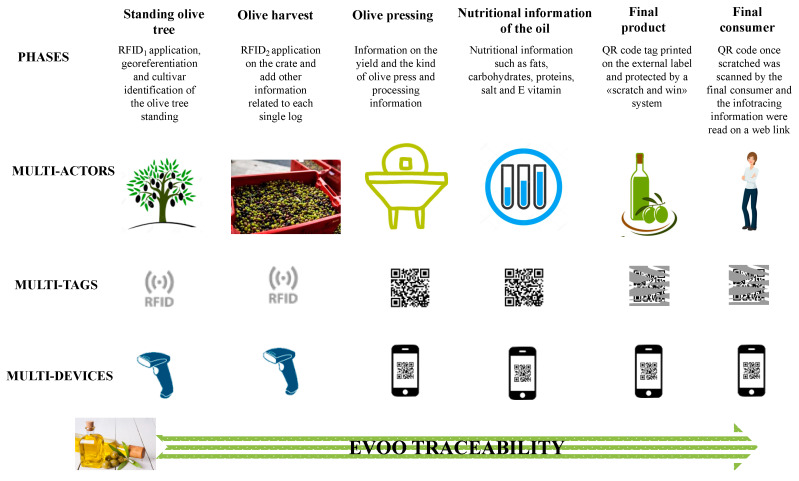
Extra virgin olive oil (EVOO) traceability scheme and phases including relative multi-actors, multi-tags and multi-devices. The standing olive tree phase included application of the first radio frequency identification (RFID1); cultivar identification; first tag association and information storage in the database. During the olive harvesting the second RFID (RFID2) was applied on the crate, and other information related to each crate were added. During olive pressing, the information related to processing and yield were added. The nutritional information of the olive oil related to fats, carbohydrates, etc., was assessed and stored On the final product was printed and applied to the bottle a label with a QR code tag protected by a “scratch and win” system. Finally, once scratched, the QR code was scanned by consumers, and the information tracked by the infotracing system was read on the web through a dedicated link that was generated.

**Figure 2:**
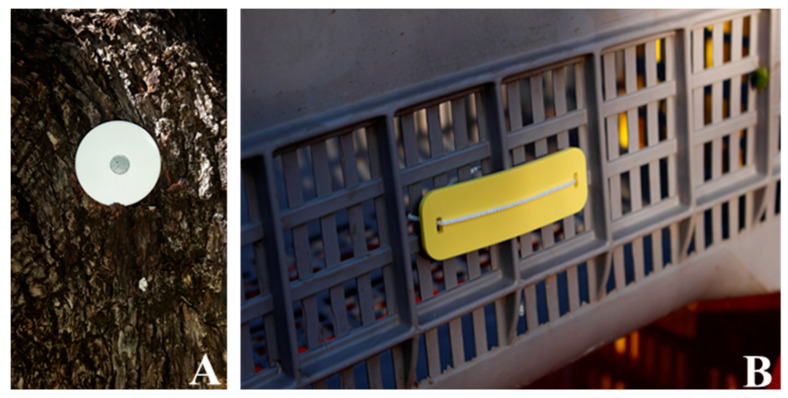
(**A**) The RFID1 standard used is UHF at 860 MHz, Class 1 Gen 2, waterproof coin shaped with central hole; (**B**) the RFID2 is UHF at 860 MHz, waterproof flexible band with two holes at the extremities fixed with a small cord on the crate.

The EVOO production chain can be summarized in the following technological traceability system (TTS; infotracing) phases (Figure 1):

- Standing olive tree: application of the first waterproof coin shaped RFID tag with a central hole (RFID1) that was positioned on the trunk in the North direction and fixed with an iron nail (Figure 2A). This first tag was used to provide to the database information related to tree cultivar (Carboncella, Frantoio, Leccino), tree GPS point and other observations (notes). A total of 33 trees (11 trees per cultivar) were tagged.
- Olive harvest: application of the second kind of RFID tag (RFID2), a waterproof flexible band with two holes at the extremities fixed with a small cord on the crate (Figure 2B). The crates were used to transport the harvested olives at the olive oil mill. Each RFID2 was uniquely associated with one tree, thus preserving the association with the RFID1 information applied in the previous phase. Here were added the following information related to each crate: crate weight, olive maturity (high, medium, low), olive defects (high, medium, low) and other information (notes) and type of harvesting (mechanized, semi-mechanized, hand harvesting). In the present work the harvest was entirely semi-mechanized for all the cultivars.
- Olive pressing: information on the yield and type of olive oil extraction process. The extraction system used for the scope was a “Il Molinetto” compact continuous oil system from Pieralisi group (Ancona, Italy) and a two-phase centrifugal separator from Alfa Laval (Monza, Italy).
- Nutritional information of the olive oil printed on EVOO bottle label such as fats (saturated, monounsaturated, polyunsaturated), carbohydrates, proteins, salt and vitamin E.
- Final product: The QR code tag was printed on the external EVOO bottle label and protected by a “scratch and win” system. The “scratch and win” system is a gamification method that protects the QR code in order to allow the consumer who buys the product to obtain the reward. The Infotracing platform was implemented with a blockchain system. Once the consumer purchases the product, scratching the label and framing the QR code with a smartphone, he or she can receive, along with the product information, a digital token of €0.05, produced by the blockchain itself, which can be spent on the reference platform. The blockchain system ensures that the information inserted along the entire supply chain is tamper-proof [6].
- Final consumer: The QR code, once scratched, was scanned by the final consumer, and the infotracing information was read through a web link.

The point of strength of the proposed TTS system mainly regards the possibility to carry information of any type (specific nutritional, DNA, farm related, etc.) in a safe way (from the standing tree to EVOO bottle). A limited production of bottles was used for experimental and demonstration purposes (100 bottles containing 500 mL each). Four kinds of EVOO were produced: three monocultivars (Carboncella, Frantoio, Leccino) and one blend (PDO Sabina).

### 2.2. RFID Technology

The open source prototype system based on RFID technology was created adopting different wireless communication protocols for each phase along the Infotracing flow. The tags were read during each of the above mentioned phases through the RFID antennas and in addition, being in an outdoor in-field context, through a portable customized reader. The codes were subsequently sent to a smartphone via Bluetooth that, using a specifically programmed Android app (Infoliva, see description below), stores the code and the related additional information entered by the operator. The customized portable reader was developed using open source technologies consisting of an Arduino^®^ micro controller unit (MCU) with an integrated Bluetooth (Bluno Mega 2560) and an RFID antenna interfaced through a serial communication port. A detailed description of the antenna is reported in Figorilli et al. [16]. The RFID standard used was UHF at 860 MHz. The tags used were Class 1 Gen 2 that, compared to the predecessors Class 1 Gen 1 that did not contain any kind of support for the processed data security, support two systems useful for the processed data security such as the TID code (the serial tag ID) and the access/kill passwords.

### 2.3. App (Infoliva) for EVOO Traceability

The app (Infoliva) for the EVOO traceability data collection was developed to support the in-field operation from standing trees to olive oil mill, providing the operators with a simple and easy-to-use tool for smartphones. The information registered through the app is stored on an internal database and subsequently synchronized on a remote server. In addition, the app uses the smartphone components and sensors (i.e., GPS and Bluetooth) to collect precious data. The GPS receiver was used to collect the trees’ geographical positions. The Bluetooth device was used for the connection between the smartphone and the external customized portable RFID reader. Internet connectivity was needed for the synchronization on the remote server; this operation can be achieved both in-field (if the internet connection is available) or afterwards, when field operations are concluded and the internet signal becomes available.

**Figure 3:**
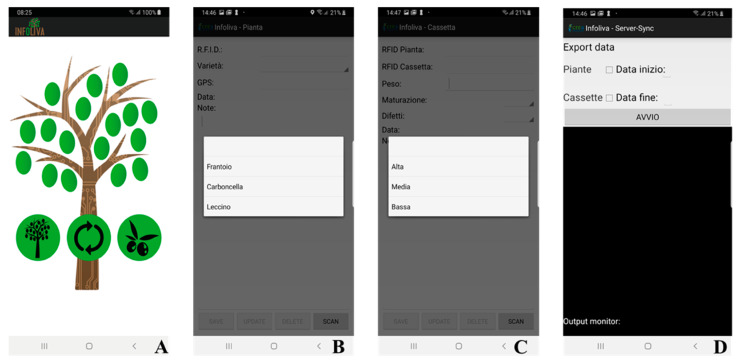
(**A**) Main activity of the Infoliva app (written in Italian to be used by local operators); (**B**) activity regarding the standing olive tree cultivar (Carboncella, Frantoio, Leccino), GPS (automated inserted and unmodifiable), date (data; automated inserted and unmodifiable) and notes; (**C**) activity regarding the olive harvest phase crate weight, olive maturation (high, medium, low), olive defects (high, medium, low), date (data; automated inserted and unmodifiable) and notes; (**D**) activity regarding the synchronization phase with the remote server and blockchain. The app was developed to be used by Italian users and could be easily modified in other languages and including other fields.

The app (Figure 3A) was predisposed to rapidly operate choices such as the cultivar identification phase or the olive harvest phase and remote synchronization phase.

Each activity regarding the Infoliva app is described below and represented in Figure 3:

- Main activity: the start page allows one to select the phase to be executed (standing olive tree or olive harvest).
- Standing olive tree (Figure 3B): through the “scan” button, the Bluetooth connection of the smartphone to the customized portable RFID reader is achieved. The device is in a waiting state ready to receive the tag RFID1 unique code that identifies the tree. When the RFID code is received, the app checks for the presence of the code. If it is an existing code, all data will be loaded and can be modified up to 2 h, while if it is a new code, the fields will be activated to enter the following information: cultivar (Carboncella, Frantoio, Leccino), GPS (automated inserted and unmodifiable), date (data; automated inserted and unmodifiable) and notes. Once inserted, the data will be saved on an internal database. Data updating and deletion functions have also been implemented and are available up to 2 h.
- Olive harvest phase (Figure 3C): as reported above, the scan button allows the reading of a RFID tag via Bluetooth. The procedure consists of the following: (1) reading the RFID1 positioned on the tree that needs to be harvested, (2) association of the plant tag to the related crates where the olives are collected, (3) adding the following information for each crate: crate weight, olive maturation (high, medium, low), olive defects (high, medium, low), date (data; automated inserted and unmodifiable), notes. Control procedures to verify the data consistence and coherence were implemented. As above described, data update and delete functions were also implemented and are available up to 2 h.

**Figure 4:**
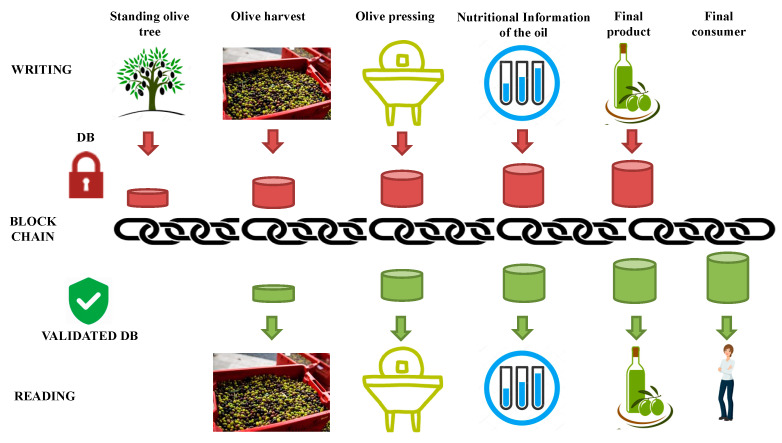
A schematic description EVOO traceability with implemented the blockchain data flow.

The whole EVOO supply/processing chain information, from standing trees to consumer, described in Section 2.1, was implemented in a personal cloud database. The standing tree and in-field information were inserted with the dedicated devices mentioned above; the other information (yield after pressing, nutritional information) were manually inserted by the operators along the chain through a web interface. The database and the architecture were built in order to be easily implemented with blockchains. A schematic description of the blockchain data flow is summarized in Figure 4. This figure, independently from the kind of platform used, could represents a typical blockchain integration. For example, the remote synchronism (Figure 3D) manages the synchronization with the remote server and the blockchain. It is activated by the following two steps: the first, before the in-field operations (olive tree marking or olive harvest), allows one to proceed with the operation once the activation code provided by the authoritative institution has been inserted; the second, the information acquired is shipped (during, if internet connection is available, or at the end of the specific in-field operation), specifying the temporal interval and the type of data (i.e., crate management).

**Figure 5:**
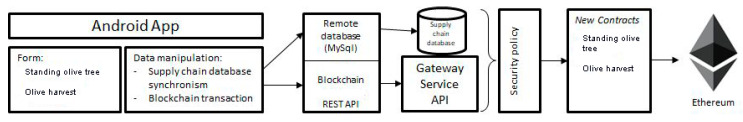
Interconnection diagram between the INFOLIVA app developed and the blockchain.

The software architecture was developed considering the integration with the blockchain. In detail, a data manipulation software layer was added to the synchronization part with the central server (the supply chain database) to make it compatible with the eventual contract to be implemented in the blockchain. In addition to the data manipulation software was added a connection section to the Gateway service API (application programming interface) to communicate with the blockchain (i.e., Ethereum). Through the “security policy” element (Figure 5), it is possible to implement parallel mechanisms of consolidation and coherence of information before the permanent validation and storage within the blockchain (Figure 5), in relation to the information that was managed via app.

### 2.4. Econometric Analysis

Besides the technical feasibility of the TTS applications and practical potential achievable benefits, an economic evaluation was performed to underline the cost impact of such a system on the olive oil production chain and thus to assess its economic sustainability. Indeed, a traceability enabled production system provides information and safety levels that potentially attract additional consumers to pay a price for the technological integration. Where consumers, and therefore producers, are willing to pay this cost, the technology can be implemented. The econometric analysis began with the analysis of part of the production process. This was represented by the final phase of olive harvesting and transfer to the oil mill, where the TTS was directly involved.

This production phase of the process was technically and economically analyzed by studying the working times and estimating the costs associated with the olive harvesting process and those relative to the TTS application, from the standing olive tree to the olives delivered to the mill. This was done to evaluate the economic impact on the final product obtained, which is EVOO.

**Figure 6:**
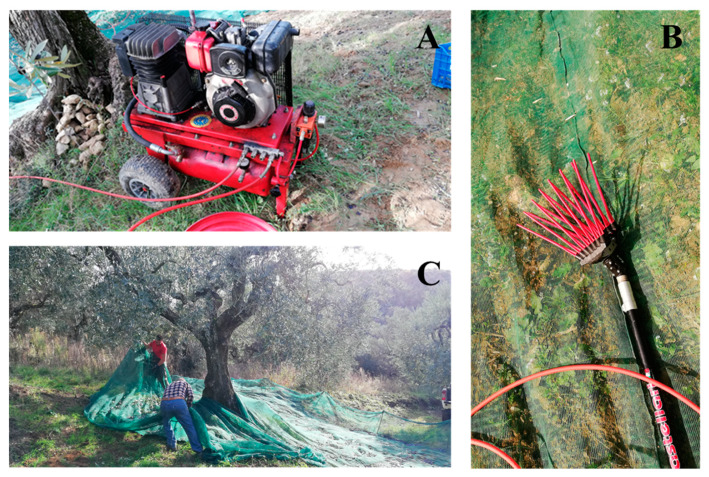
(**A**) Air compressor with 5.5 kW diesel engine; (**B**) pneumatic combs for olive harvesting; (**C**) laying nets for the interception of olives on the ground.

Regarding the olive harvesting process, the current and most used working system in the PDO Sabina oil area under study was observed. The harvesting technique applied was semi-mechanized [19], with the use of handheld pneumatic combs driven by an air compressor powered with a diesel engine (Figure 6A).

The working team was made up of four operators; two of them assisted with the harvesting with the use of 3 m rods with combs (Figure 6B), and the other two were assigned to the movement and positioning on the ground of the olive interception nets (Figure 6C), and additionally to the loading of the olives into crates before transporting them to the oil mill.

Work times were recorded in a plot of the reference farm of approximately 5500 m^2^, on which there were 60 olive trees of the “Frantoio” cultivar, whose average age is about 90 years. A data set was built for each olive tree harvested, detecting the diameters, the productions obtained and the number of crates used (Table 1). For each tree examined, the working times necessary to complete the olive harvesting process were recorded. Along with these assessments was also recorded the time necessary for the application of the TTS on every single tree considered. This info were used to estimate the economic impact that these technologies could entail if embraced by an olive oil producer.

The time study was oriented to determine the productive working time with respect to a single tree harvested. The time elements considered were the following:

- arrangement of the nets under the tree to be harvested (CT): the work was carried out by 2 operators and included the drafting of 2 or more nets per tree in order to best cover the area where the olives will fall;
- harvest time (HT): represents the work time necessary to detach the olives from the tree; it was carried out by 2 operators by means of the pneumatic combs from the ground;
- olive accumulation time (AT): represents the time necessary to remove the nets from the harvested plant and gather the olives together in a point of the net before cleaning the twigs and leaves;
- cleaning and unload time (UT): represents the time necessary for a gross cleaning from twigs and leaves of the olives collected and the unloading of the same in the crates;
- delays (D): the whole time needed for mechanical delays as needed for service or machine repair, and personal delays as interruptions due to the operators, including rest breaks;
- net productive time (NT): represented by the sum of all precedent elements without D;
- gross productive time (GT): obtained by the sum of NT and D.

The work times were detected using a Minerva chronometric table with three centesimal chronometers, applying the snap-back timing method and making videos for further checking of the work times observed for each olive harvesting phase.

The time study, distinguished for each work element, was observed with respect to the single olive tree. A set of data was constructed correlating the work time with the olives harvested. Statistical analyses were performed using the SPSS 18 statistical package (IBM, Armonk, NY, USA).

Based on the GT values of the olive harvesting process for each olive tree, the gross productivity (GP) was determined applying the following formula:

GP = 60·P/GT

where GP is the olive harvesting work productivity of the yard expressed in kg per hour (kg h^−1^); P is the olive production expressed in kg per tree (kg tree^−1^); GT is the gross productive time, expressed in minutes per tree (min tree^−1^).

The productivity was also calculated as

GPt = GP/P

where GPt represents the trees harvested per hour (trees h^−1^); GP is the hourly productivity obtained by Formula (1); P is the olive production per tree (kg tree^−1^). 

On the basis of the productivity values, the relative harvesting operation cost per tree was calculated. The yard hourly cost was calculated with analytical formulas considering fixed and variable costs regarding machinery and equipment [19,20], together with the labor cost. Table 2 shows both the main technical and economic elements considered, and the result of the calculation that led to a value of the yard hourly cost equal to 63.31 € h^−1^ based on four operators, a motor compressor and two rods with combs.

In addition to the costs of the work yard, also calculated were the depreciation costs, interest (4%) and overhead (3%) for the nets and crates considering eight years of useful life. The number of 60 crates ha^−1^ and 8 nets (measuring each 10 m × 5 m) were considered for a total investment cost of 320 and 300 € ha^−1^, respectively; the depreciation charges calculated were 0.36 and 0.34 € tree^−1^, respectively. The parameters considered for the economic evaluation were the total cost per tree and the cost per kg of olives, using the formulas expressed below.

The first formula is the following:

Ct = Ch/GPt(1)

where Ct represents the cost per tree (€ tree^−1^); Ch is the hourly cost of the harvesting yard (€ h^−1^); GPt is the productivity obtained by Formula (2).

The cost per olive production unit was calculated using the following formula:

Cp = Ct/P

where Cp is the cost per kg of olives (€ kg^−1^); Ct is the cost per tree obtained by Formula (3); P is the olive production per tree (kg tree^−1^).

The cost per EVOO production unit (€ kg^−1^) was calculated, with respect to a single tree, using the following formula:

Co = Ct/Po

where Co is the olive oil cost (€ kg^−1^); Ct is the cost per tree, according to Formula (3); Po is the olive oil production per tree (kg tree^−1^).

For the cost calculation of the TTS application within the olive supply chain, and for the evaluation of the impact on the final product, the elements reported in Table 3 were considered.

The purpose of the work is to identify the economic convenience limit of the traceability process in relation to what the consumer is willing to accept as a possible EVOO market price surplus, knowing with certainty that the product is fully traced and precisely identifiable, and additional information useful for food safety purposes is available. The TTS assessment methodology considered what was proposed by other authors applied to different sectors [6,16,21], which was based their analysis on the four following phases:

- Implementation of all the components necessary to set up the TTS (tag, reader, software, etc.), with the determination of the initial capital to be invested to set up the system.
- Application and use of the TTS assessing all the operations necessary for regular operation; detection of current costs to apply the tags in the traced objects (standing trees, crates of filled olives, derived oil), which must be marked along the production chain for complete traceability and the transfer of the stored data.
- Implementation and modernization of the TTS for receiving and reading the information stored in the field, in the farm and in the oil mill. The required investment amortization must be spread over several years of activity.
- Evaluation and verification of the benefits that the TTS application can generate within the production chain. Assessment of the economic convenience derived by the technology used.

With reference to the case study, the judgment of economic convenience of the TTS application is based on the calculation of the increase in cost generated by the TTS during olive harvesting, processing and transport to the mill, in comparison to the costs normally incurred in the absence of its application. The analysis was carried out by developing correlation models of these costs according to the level of olive production obtained for each tree. The judgment of convenience was made in relation to the limit of potential acceptability of the percentage increase of the EVOO sale price on the market attributable to the technological application.

## 3. Results and Discussion

### 3.1. Technological Traceability System

The investigations conducted in the olive grove, after 1 year from their application, showed that 31 tags positioned on the 33 olive trees belonging to the three cultivars (Carboncella, Frantoio and Leccino) were correctly read by the dedicated app and therefore proved to be functional, recording all the necessary information. Only two labels could not be read because of the nail due to the fact that they had been repaired, removed and the label was peeling off the bark of the tree. Each tag uniquely associated with a tree was associated with crates. For each plant, the following information relating to each crate was added: weight of the crates, maturity of the olives (high, medium, low), defects of the olives (high, medium, low), other information (notes) and type of collection. For the three cultivars (Carboncella, Frantoio and Leccino), 641.5 kg, 341 kg and 134 kg of olives were collected, respectively. The weights of the crates of olives were recorded in the mill on the day of collection; the following day, the olives of three cultivars (Carboncella, Frantoio and Leccino) were pressed and the yield was calculated (17.20 kg, 16.27 kg and 20 kg, respectively). Subsequently, nutritional information on the oil was carried out by measuring the fats (saturated, monounsaturated, polyunsaturated), carbohydrates, proteins, salt and vitamin E. The information acquired was successfully recorded by TTS infotracing. At that point, through the QR code tag printed on the external label of the 100 EVOO bottle and protected by a “scratch and win” system, consumers were able to access information regarding the entire EVOO supply chain examined. This system is a gamification method that protects the QR code to allow the consumer who buys the product to get the prize. The infotracing platform is implemented with blockchain. Once the consumer purchases the product, scratching the label and framing the QR code with a smartphone, he or she receives, together with the product information, a digital token of €0.05, produced by the blockchain itself, which can be spent on the platform of reference. The blockchain system allows one to ensure that the information entered along the entire supply chain is tamper-proof. In this case, innovation is represented not only by the introduction of the blockchain, but also by the combined use of the “scratch and win” system to protect the consumer. The system includes standard motivational models and technological models within the reach of the consumer [6]. 

### 3.2. Economic Analysis

The economic results obtained for the olive harvesting are comparable with those conducted with the same operating conditions but by applied during the production process, namely the RFID traceability technology. The aim was to highlight the greater charges necessary to apply the innovative technological system in-field, highlighting the level of acceptability, sustainability, usefulness and any advantages deriving from the introduction of this technology within the olive oil production chain. The results must be compared with the consumer perception of a greater transparency of the product, through a safe certification of its origin and provenance, as well as a guarantee of higher food safety and quality standards of the marketed product generated by the TTS.

The sample of 60 olive trees observed during the harvesting operation recorded a total olive production of 1855.5 kg, corresponding to 30.92 ± 15.85 (mean ± SD) kg tree^−1^.

The overall time taken by the yard for the harvesting operation was 1047.18 min, corresponding to 17.45 ± 3.26 (mean ± SD) min tree^−1^. 

**Figure 7:**
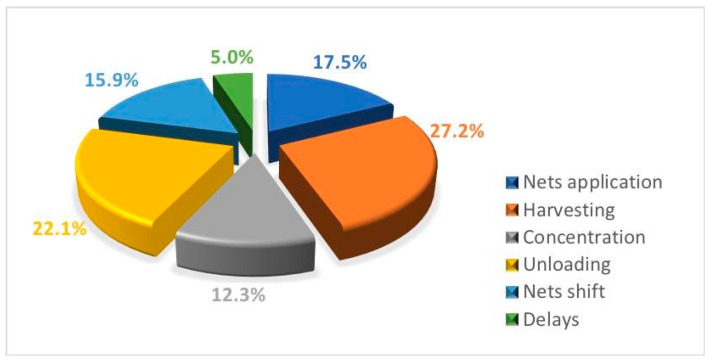
Percentage breakdown of working times in the different phases of olive harvesting.

The percentage breakdown of working times in the different phases is shown in Figure 7.

**Figure 8:**
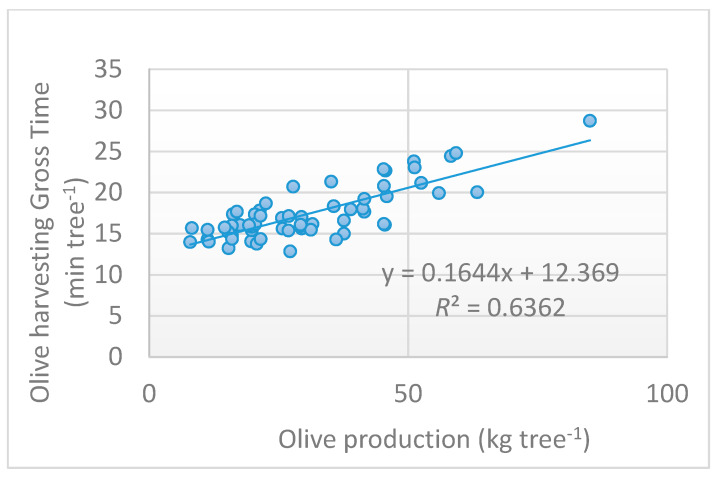
Olive harvesting gross time per tree expressed as a function of olive production.

The olive harvesting was the most time-consuming phase, requiring 27.2% of the total time, followed by the olive unloading phase (22.1%). The movement of the nets, represented by net application, concentration and shifting, required 17.5%, 12.3% and 15.9%, respectively, of the time, or an overall total of about 46% of the time. The delays were very limited and took up just 5% of the total time. Figure 8 represents the relation between the harvesting gross time for tree and olive production. The determination coefficient (*R*^2^) of the equation was equal to 0.636, which indicates a good correlation between working time and tree production, with high statistical significance based on a *t*-test (*p* < 0.001).

**Figure 9:**
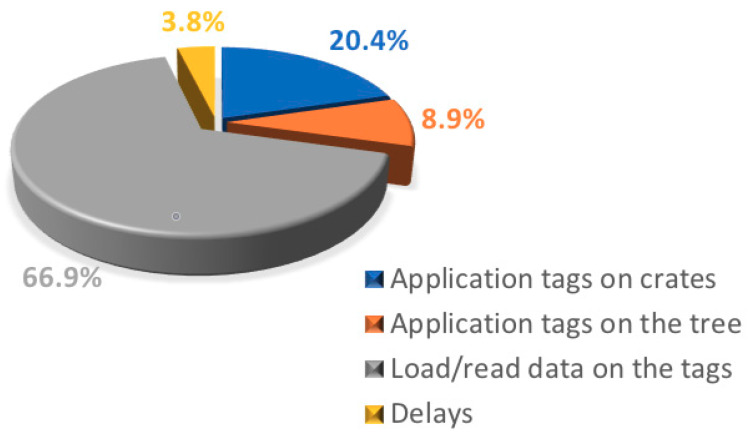
Percentage breakdown of the technological traceability system (TTS) application times in the different phases.

Figure 9 shows the percentage breakdown of the gross time consumed for the TTS application. The time consumed for loading data and for application of the tags on the stand trees and on the olive crates represented about 67% the total. The total time consumed for TTS application was 265.5 min, corresponding to an average of 4.42 (±1.40) min tree^–1^.

**Figure 10:**
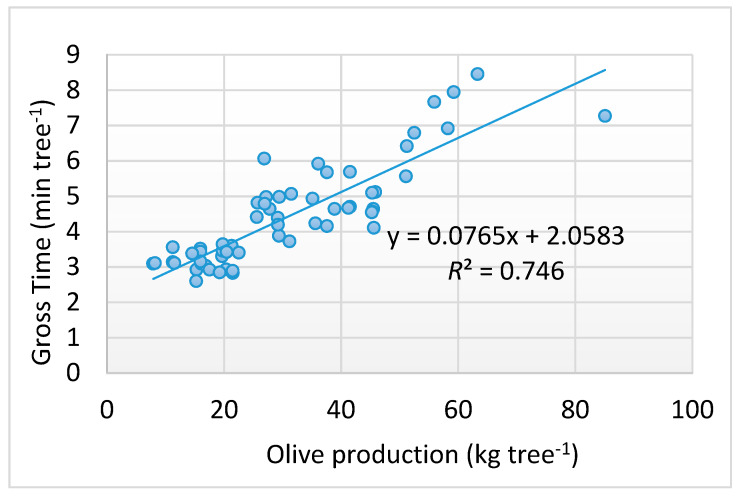
Relationship between gross time of the TTS application and olive production per tree.

Figure 10 reports the linear regression of the dependent variable (gross time) with respect to the independent variable (olive production) showing high correlation (*R*^2^ = 0.746) along with the statistical analysis indicating a highly significant value for the *t*-test (*p* < 0.001). In addition to the specific time dedicated to the TTS application, also considered was the increased time spent for unloading the olives in the tagged crates for each tree, which were very often not filled up. This time amounted to 126.96 min, which was 2.12 (±0.80) min tree^−1^.

**Figure 11:**
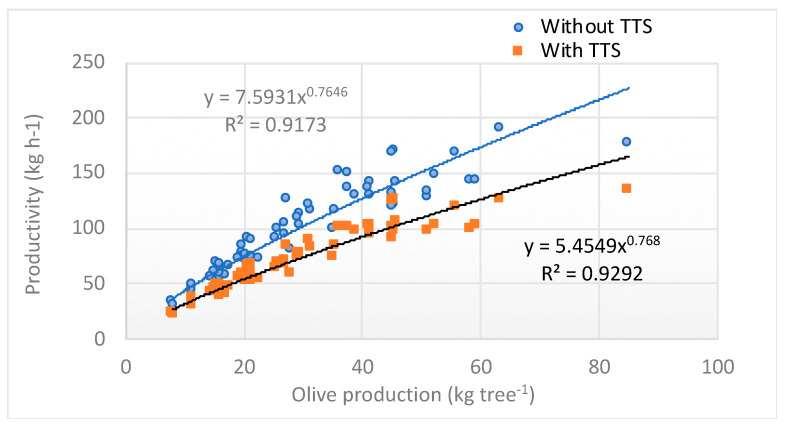
Productivity of the olive harvesting yard, with or without TTS application, as a function of olive production per tree.

Considering the time spent on the TTS application, the result was a reduction in the productivity of the olive harvesting yard, as reported in Figure 11, which shows the variation in productivity as a function of the olive production per tree in the two mentioned cases (with and without TTS). As a rule, the TTS application involved a reduction in the productivity in function of the olive production per tree, with an average reduction per tree of approximately 28.2%.

**Figure 12:**
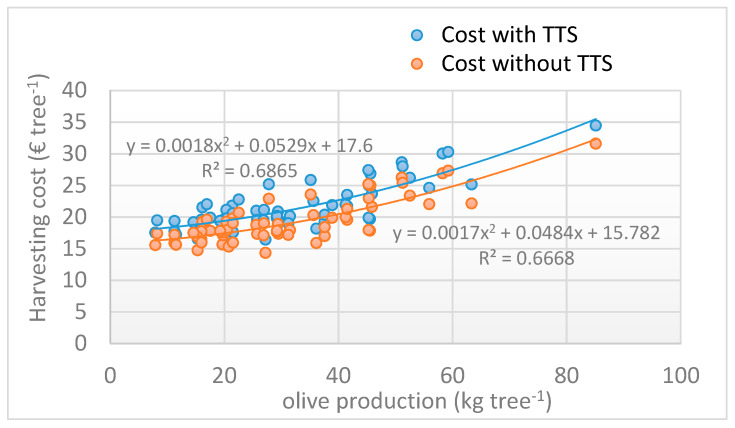
Harvesting cost with and without TTS application as a function of olive production per tree.

**Figure 13:**
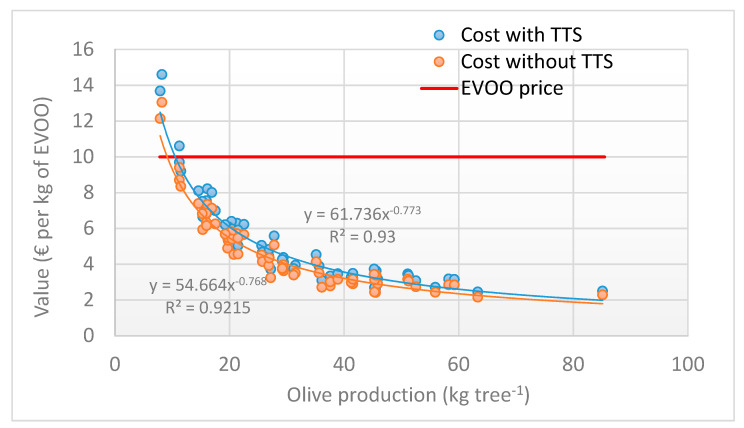
Comparison between harvesting cost, with or without TTS, and the EVOO market price as a function of olive production per tree.

**Figure 14:**
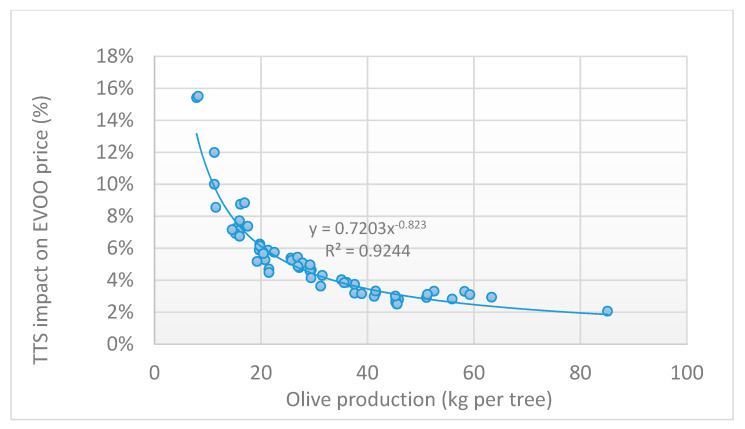
Percentage impact of the TTS application on the EVOO market price as a function of the olive production per tree.

The economic results of the analysis carried out are summarized in Figure 12, Figure 13 and Figure 14. Figure 12 reports the cost of the harvesting yard per tree, with and without TTS application, in the process of olive production. The trend of the two cost curves shows an increase in function of the production per tree. Considering the TTS application, the cost varied from a minimum of 16.42 € tree^−1^ to a maximum of 34.44 € tree^−1^, with an average of 21.46 (±3.78) € tree^−1^.

Figure 13 compares the costs per kg of oil produced per tree (yield of olives in oil equal to 16.73%) with the EVOO market price. For very low olive production, the costs increased to the point of exceeding the olive oil price, highlighting the poor economic sustainability of both curves for production lower than 9–10.5 kg tree^−1^. Below these values, the impact of the TTS application also became heavier, reaching an incidence of over 10% (Figure 14). For olive production exceeding 30 kg^−1^, this incidence fell below 5%, making the TTS application highly sustainable.

A study by Violino et al. [6], evidenced how the consumers are becoming very attentive and inclined towards new technologies that can guarantee traceability, willing to spend up to 17.8% more than the conventional sale price for one bottle of EVOO. This means that with regard to the results obtained, even when the incidence of the TTS application is higher (15.5%; i.e., low olive production), this remain below the reference percentage mentioned above. The impact on the potential price increase of the olive oil due to the application of the TTS is therefore economically sustainable and acceptable to the final consumer. Moreover, considering an average production per tree such as the one examined, the incidence of the TTS application drops below 5%, making this surplus cost negligible and highly sustainable.

As reported by Violino et al. [6], the implementation of the QR-B traceability system at the agricultural production level has a more significant percentage of cost increases for the EVOO per bottle (27.12%), while by consortium and industrial levels, the percentage costs decrease considerably, with values equal to 2.01% and 0.03%, respectively. Therefore, for these production levels, the QR-B system always appears below 17.8% (the additional price that consumers were willing to pay, for the integration of TTS infotracing).

The proposed TTS infotracing system can potentially bring benefits to both consumers and producers. Generally speaking, the consumer will be able to obtain EVOO full traceability and additional information. This system represents an excellent solution to guarantee reliability, transparency and security, in particular against fraud [6]. The information flow can be tracked by RFID and the additional aid of a blockchain system. Although such technologies potentially offer very trustable levels of safety against the possibility to modify the inserted data, there is always the possibility to physically counterfeit the products along the chain or, for example, to insert fake information in first place that will be encapsulated and carried along the entire chain by the blockchain system [16]. The potential impact of the proposed system, as always with these technologies, is to try to reduce as much as possible, but unfortunately not to eliminate, the possibility to fake the information with minimal additional economic impact value (the possibility to add useful information, e.g., for product storytelling). Moreover, the TTS can be potentially useful to identify punctual points to characterize and control the products through analytical DNA-based methods. This may lead to a reduction of their random use. The producers that choose to implement a traceability system almost certainly want to demonstrate to the consumers that their products are authentic and produced, transported and sold in a defined amount and within a certain time. This ensures the consumer that the products have not been re-labeled with different data. A forger is unlikely willing to implement a technology that can reduce (even if not to eliminate) potential fraud. Thus, the TTS is partially based on a trust between producer and consumer.

Indeed, on one hand, the producer gains and archives georeferenced information relative to his or her olive orchard that is useful to improve its management strategy in light of a precision farming philosophy (e.g., optimizing a site-specific fertilization plan). On the other hand, the consumers of course benefit from the traceability information reported on the final product. Note that the information that is readable, e.g., on the bottles, is representative of a set of trees in a given area significative for a certain orchard. As mentioned above, producers could obtain several advantages in terms of information helpful for the following precision management strategies [22] regarding:

- Production yield;
- Pruning indications;
- Pests and diseases/treatments;
- Fertilization;
- Irrigation.

As a further result, the net value of the orchard could increase on the market due to the historical information on the management of each single tree.

Another advantage owing to this traceability system is related to the manufacturer. Indeed, thanks to the use of blockchain systems, it represents a strong tool to guarantee the EVOO origin. As a result, a manufacturer could promote his or her product with marketing strategies that, on the one hand, provide a guarantee to the consumer, and on the other retain consumer loyalty to the company, which in turn can get a higher price, given the guarantee obtained. As Violino et al. [6] pointed out, the consumers are very attentive and inclined towards the new traceability technologies of EVOO and are willing to pay up to the 17.8% more than the conventional sale price.

## 4. Conclusions

The present work examines the potential implementation impact of the application of a TTS, in comparison to conventional management, on an EVOO production system relying on a semi-mechanized harvesting system. The application of TTS showed good possibilities for implementation in terms of estimated surplus cost the consumer would be willing to pay for its implementation. The traceability system implementation provides information at different levels that are potentially helpful for the farmer, manufacturer and consumer. The economic analysis shows higher cost for the present farming system; however, its adoption is potentially viable and will score further benefits considering a farming system opting for a higher level of mechanization for olive harvesting, such as the use of a trunk-shaker equipped with a collecting umbrella. This mechanization level is typical of a farm of a larger scale. Indeed, the use of this machine, with a driver and an operator on the ground, can raise the productivity and reduce the harvesting costs by up to 50% in comparison with those examined in the present paper [19]. Additionally, the application costs of the TTS would decrease due to the lower times needed to load the tagged crates. By using this higher level of mechanization, the average incidence of the TTS application costs on the EVOO final price would be below 4%, making the application of this technological innovation even more sustainable. Thus, larger farm sizes with higher mechanization levels receive higher benefits from TTS implementation, which would have a lower economic impact. Another important and interesting aspect, which could be implemented in the future, is related to the possibility to remotely detect the minimum and maximum yields of a production area by using remote sensors and drones or other aerial vehicles to acquire images to process. The introduction of these monitoring systems could perfectly be integrated in a traceability system for fighting counterfeits related to the oil sector. This could be done by counting plants and estimating the production on the base of several parameters extracted by 3D or 2D reconstructions. The information would be stored along the chain given the presented technology suitable for the scope.

## Figures and Tables

**Table 1:** Main characteristics of the olive trees observed (“Frantoio” cultivar).

Parameters	Diameter (cm)	Olive Production (kg)	Crates (*N*)
Average value	50.67	30.9	1.73
Standard deviation	4.07	17.46	0.73
Minimum value	43.20	7.89	1.00
Maximum value	60.10	85.10	4.00

**Table 2:** Main elements considered in the cost calculation and hourly cost of the olive harvesting yard.

Costs	Motor Compressor and Pneumatic Combs
Purchase price (€)	2500.00
Salvage value (€)	313.00
Service life (years)	8
Productive time (h year^−1^)	480
Power (kW)	5.6
Interest rate (%)	4.0
Fuel consumption (L h^−1^)	1.14
Lubricant consumption (L h^−1^)	0.02
Labor cost (€ h^−1^)	15.00
Machine cost (€ h^−1^)	3.31
Workers (*N*)	4
Harvesting yard cost (€ h^−1^)	63.31

**Table 3:** Elements for calculating the technological traceability system (TTS) application cost. As for crates, radio frequency identification (RFID) lifespan on standing trees was considered to be 8 years.

Element	Values
RFID price (€)	0.10
RFID unit cost (€ for each per year)	0.0125
Reader price (€)	150
Reader cost (€ tree^−1^)	0.46
Software cost	open source
Data management (€ h^−1^)	15
